# Synthesis, characterization, and anti-corrosion properties of an 8-hydroxyquinoline derivative

**DOI:** 10.1016/j.heliyon.2019.e02895

**Published:** 2019-11-26

**Authors:** Zahra M. Alamshany, Aisha A. Ganash

**Affiliations:** Chemistry Department, Faculty of Science, King Abdulaziz University, Jeddah, Saudi Arabia

**Keywords:** Electrochemistry, Organic chemistry, Physical chemistry, 1,3-bis(quinolin-8-yloxy) propane, Polarization, DFT, EIS, NMR, FTIR

## Abstract

A new 8-hydroxyquinoline derivative, namely 1,3-bis(quinoline-8-dimethylformamide) propane (BQYP), was synthesized and characterized by different spectral methods, such as ^1^H NMR, ^13^C NMR, and FTIR spectra. The anticorrosive properties of the BQYP molecule against the corrosion of mild steel were tested in 2 M Η_2_SO_4_ acid with a varied range of concentrations (0.05–1 mM) at different temperatures using the electrochemical technique. It was clear that adsorption acted according to Langmuir's relationship. The inhibition effect improved with increases in concentration of inhibitor (~91% for 1 mM at 298 °K) and was reduced with increasing temperature. Finally, the density functional theory (DFT), with bases set according to the B3LYP/6-311+G (d,p) level, was used for calculating the quantum parameter to explain the effect of the electronic structure of the BQYP molecule on providing the experimental findings.

## 1 Introduction

**Scheme 1:**
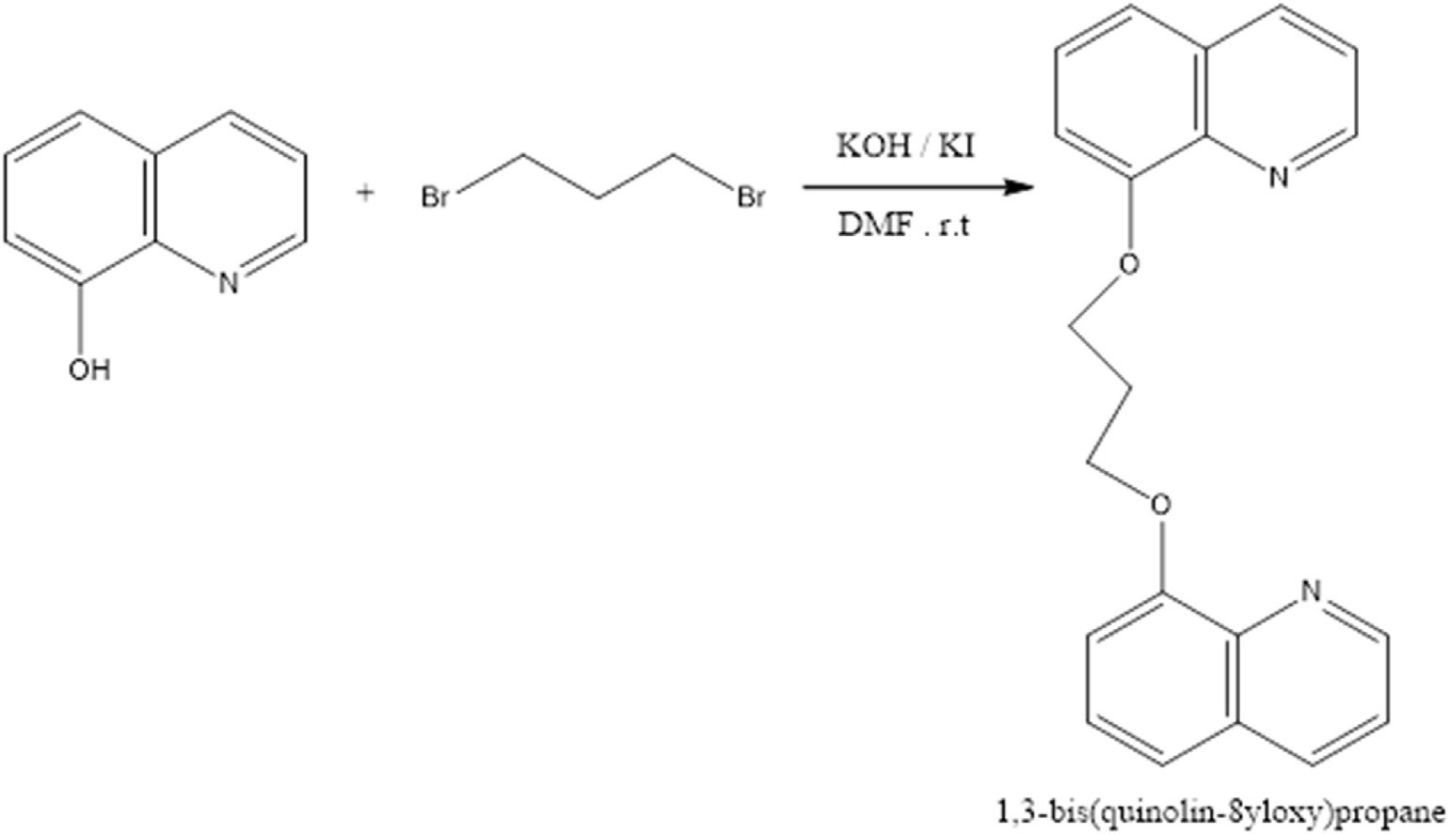
Synthesis diagram of BQYP compound.

The compound 8-hydroxyquinoline (8-HQ), one of the most popular and versatile organic compounds, is a natural crystalline material made up of two rings: a phenyl ring fused with pyridine ring. It is a monoprotic bidentate chelating agent. It is usually prepared from quinoline-8-sulfonic acid and a Skraup synthesis from 2-aminophenol. There are many applications for 8-hydroxyquinoline and its derivatives, ranging from pharmacological and pharmaceutical agents, to electron carriers in organic light-emitting diodes (OLEDs), and fluorescent chemosensors for metal ions. In addition, some derivatives have also been reported to act as organic inhibitors of corrosion (Al-Busafi et al., 2014; Liu et al., 2014; El Faydy et al., 2016, 2017; Cicileo et al., 1998; El Faydy et al., 2018; Gerengi et al., 2016). In real life situations such as factories, different problems may arise. Corrosion is considered a hazardous issue and leads to considerable losses in life, time, and effort. As is well-known, steel is the most used material in many industries, but unfortunately it corrodes in aggressive acid solutions (see Scheme 1).

Thus, many research studies have been carried out to control corrosion. The most practical approach to reduce the effects of corrosion is adding small quantities of inhibitor substance. However, Fekry et al. (Fekry and Ameer, 2010) pointed out that the addition of organic compounds with rich electron heteroatoms, such as Ο, Ν, P, and S, yields excellent results in inhibiting the corrosion of steel materials in acid solution (Ansari et al., 2014; Raja et al., 2013; Khamis et al., 2013; Abboud et al., 2012; Singh et al., 2014), although the efficiency of the inhibitory process depends mostly on the types and structures of an adsorbed film on the metal surface (Rosliza et al., 2008; Oguzie, 2005).

The present study will focus on examining the different concentrations of an 8-hydroxyquinoline derivative, namely 1,3-bis(quinoline-8-dimethylformamide) propane (BQYP), as a new prepared compound that works as a highly effective inhibitor for mild steel in a 2 Μ Η_2_SO_4_ solution at a range of temperatures using electrochemical measurement. The experimental results are provided by quantum chemical calculation using density functional theory (DFT) carried out on Gaussian-09 software to explore the relationship between the geometrical structure of BQYP and its protective ability.

## 2 Experimental

### 2.1 Materials

The 8-hydroxyquinoline and 1,3-dibromopropane were purchased from Sigma Aldrich Chemical Co. The solvents used in the present work were n-hexane, ethyl acetate (Sigma Aldrich), and DMF (Fluka). The corrosive 2 M H_2_SO_4_ solution was prepared by dilution using distilled water. Different concentrations of BQYP as inhibitors were prepared and added to the acid solution. NMR (^1^H and ^13^C) spectra were registered using a Bruker Avance (400 MHz). The progress of the reaction was followed by Thin-Layer Chromatography (TLC) using silica gel 60 F254 (E. Merck) plates.

### 2.2 Preparation of 1,3-bis(quinoline-8-yloxy) propane

**Fig. 1:**
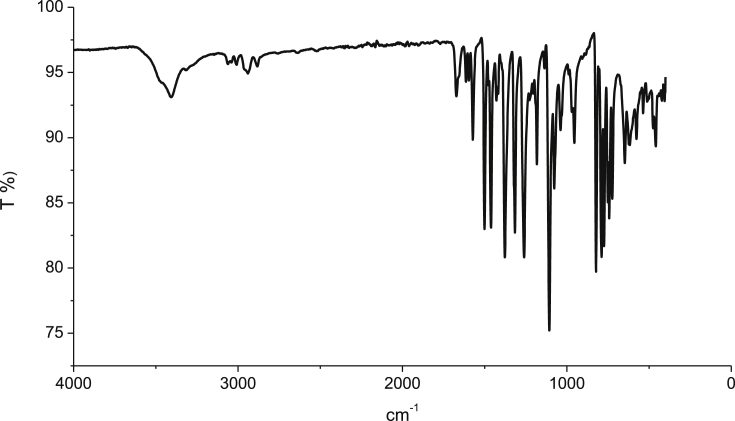
IR spectra of BQYP molecule.

**Fig. 2:**
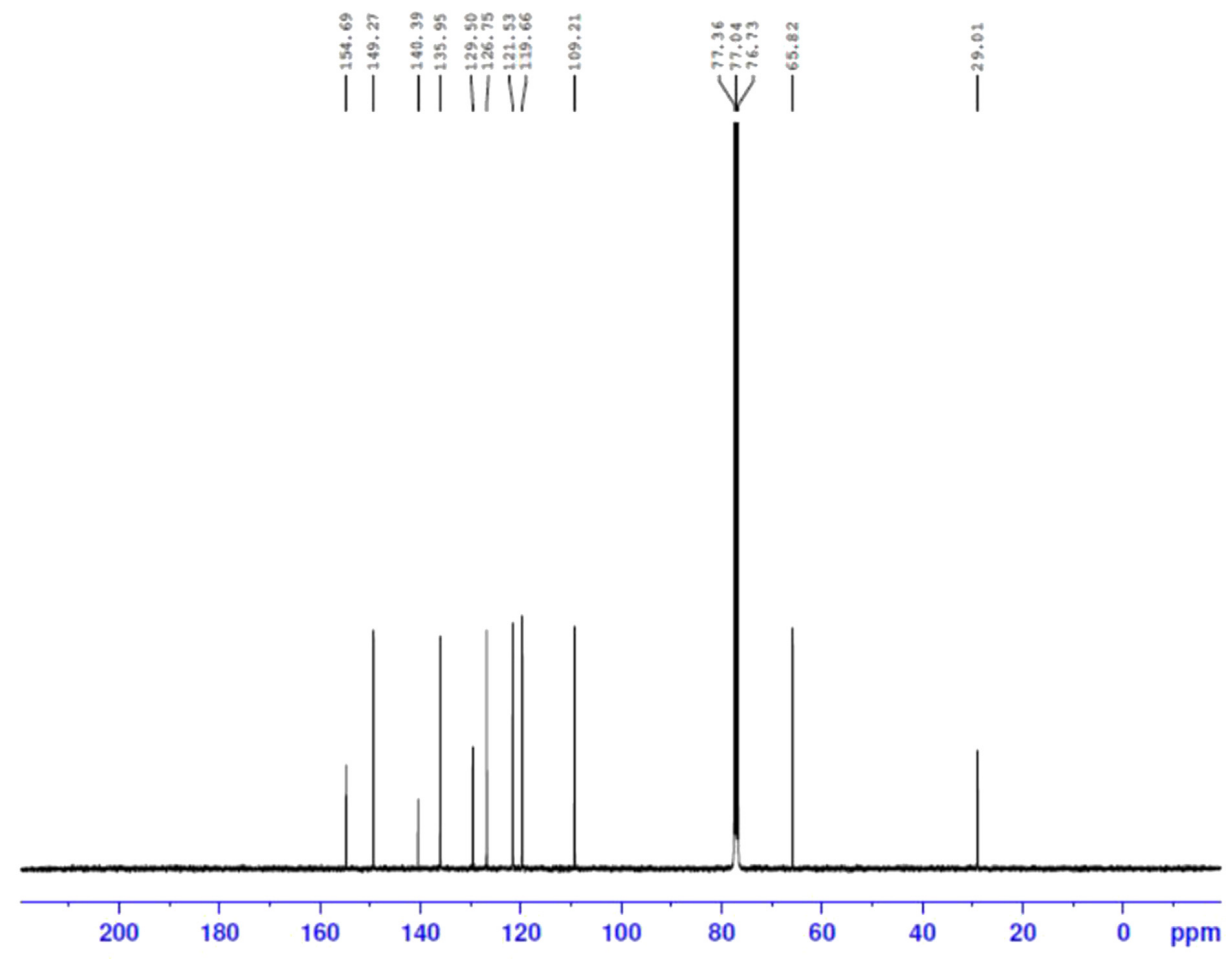
^13^C NMR spectra of BQYP molecule.

**Fig. 3:**
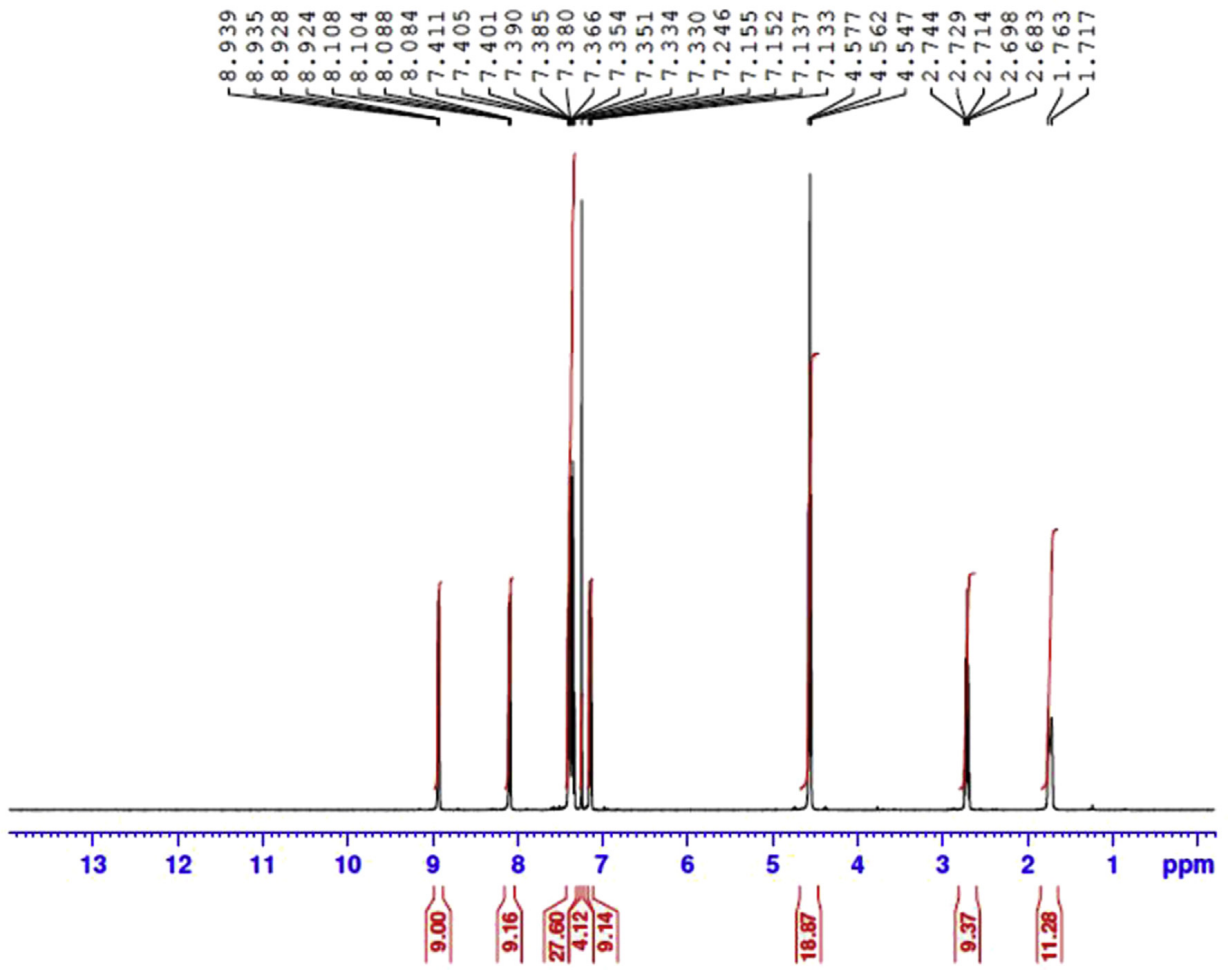
^1^H NMR spectra of BQYP molecule.

**Fig. 4:**
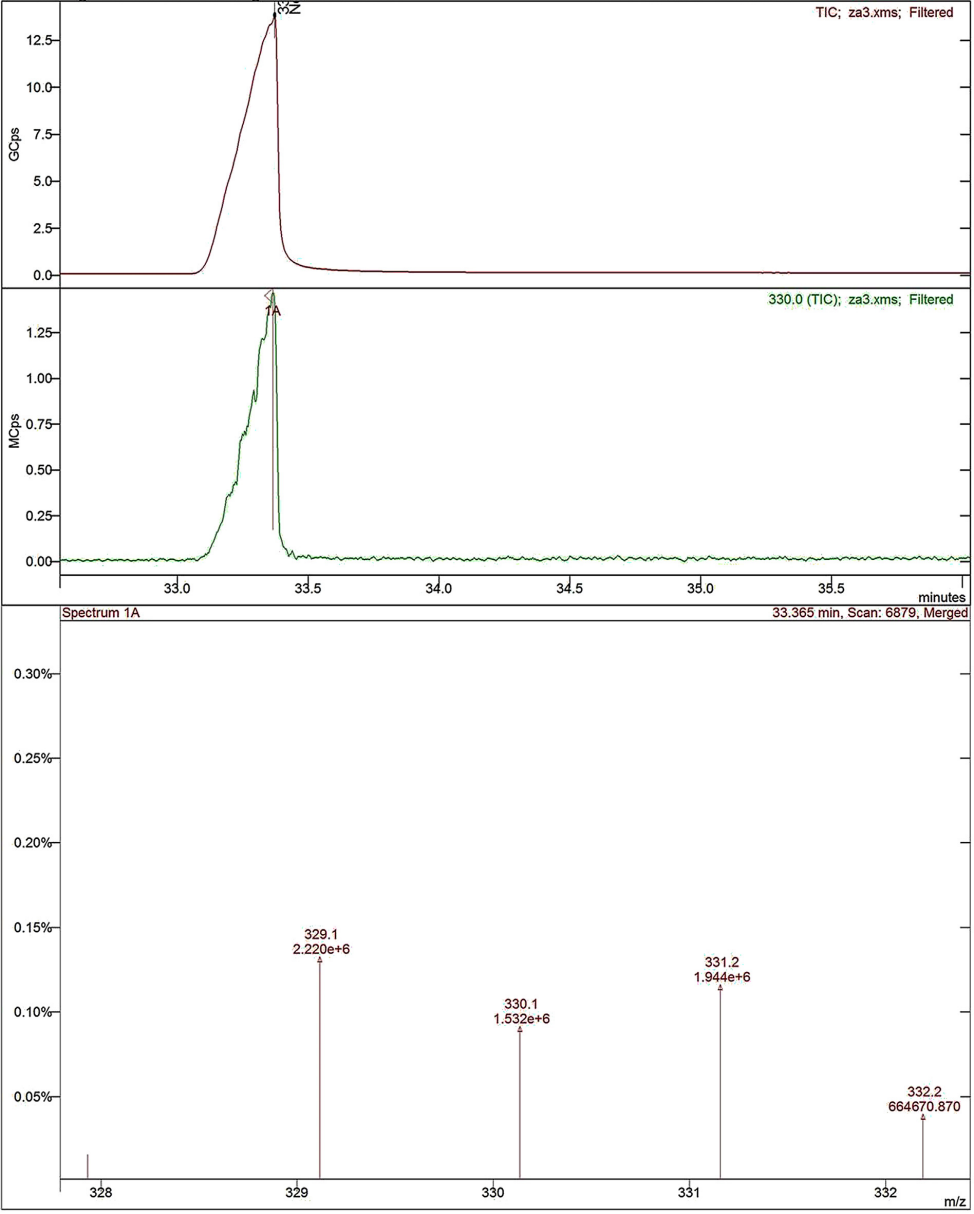
GC–MS spectra of BQYP molecule.

Potassium hydroxide (2.003 g, 0.0357 mol) was added to a solution of 8-hydroxyquinoline (3.45 g, 0.0238 mol), 1,3-dibromopropane (2.4 g, 0.0119 mol), and potassium iodide (catalytic) in 50 mL dimethylformamide (DMF) under argon atmosphere. The reaction mixture was stirred for 4–5 h at room temperature and then 200 mL of water was added. The crude product was extracted with diethyl ether (3 x 50 mL), and the organic layer was washed with saturated ammonium chloride aqueous solution and then water. The organic layer was dried over anhydrous sodium sulfate. After removing the solvent under reduced pressure, the residue was purified by column chromatography (eluent: n-hexane/ethyl acetate, 8:2) on silica gel to obtain colorless product with good yield (Katritzky et al., 2000; El-Shishtawy et al., 2017). The synthesized compound structures were confirmed by IR spectrum as shown in Fig. 1, ^13^C and ^1^H NMR spectra (Fig. 2 and Fig. 3) and GC–MS as depicted in Fig. 4.

The analytical data of the compound is:

For 1,3-bis(quinolin-8-yloxy)propane, Yield 75%; m.p. 98–100 °C. ^**1**^**H NMR** (CDCl3, 400 MHz): ∂ = 2.71 (quintet, 2H, J = 12.4 Hz, CH_2_), 4.564 (t, 4H, J = 12.4 Hz, 2OCH_2_), 7.14 (d, 2H, J = 7.6 Hz, Ar-CH), 7.33–7.42 (m, 6H, Ar-CH), 8.10 (d, 2H, J = 8 Hz, Ar-CH)), 8.93 (d, 2H, J = 4.4 Hz, Ar-CH). ^**13**^**C NMR:** 29.01, 65.82, 109.21, 121.53, 126.75, 129.50, 135.95, 140.39, 149.27, 154.69**. IR:** 1671.95, 1500.95, 1106 cm^−1^, **(m + H, 332.2)**.

### 2.3 The corrosion test measurement

The electrochemical measurement was carried out in a corrosion cell equipped with three electrodes: a steel electrode (with a wt% composition of 0.055% of C, 0.010% of S, 0.008% of P, 0.007% of Si, 0.179% of Mn and the rest is Fe) as an indicator electrode with exposed area equal to 0.970 cm^2^; a Pt sheet (5 cm^2^ length, 0.5 cm^2^ width and 0.3 cm^2^ in thickness) as counter electrode; and finally the reference electrode of Ag/AgCl (3 M KCl). The three electrodes were inserted into a polarization cell compartment with three slots. The working electrode was polished using SiC polishing papers (grade up to 1200), washed with distilled water and then with ethanol, and finally allowed to dry at room temperature. The inhibition effect of BQYP was studied by using the potentiodynamic polarization technique in a potential range of –700 to –200 mV at 60 mV/min; the other procedure used was electrochemical impedance spectroscopy (ΕΙS) in the frequency range of 30 kHz to 0.5 Hz with an amplitude equal to 10 mV.

The electrochemical measurement was conducted using a potentiostat/galvanostat ACM Gill AC single-channel instrument connected to a personal PC. All tests were conducted at 298 °K except for those examining the effects of temperature.

## 3 Results and discussion

### 3.1 Synthesis and characterization of BQYP

BQYP was prepared using a mixture of 8-hydroxyquinoline and 1,3-dibromopropane, with potassium hydroxide and potassium iodide as catalysts, in 50 mL dimethylformamide, as shown in Scheme 1. The reaction mixture was stirred for 4–5 h at r.t. and then 200 mL of water was added. The crude product was extracted with diethyl ether. The residue was purified by column chromatography to obtain a colorless product with good yield.

The ^1^H NMR nicely confirmed the two methoxy groups (OCH_2_) that appeared at 4.60 ppm and the CH_2_ observed at 2.7 ppm. The aromatic protons of the compound appeared within the range of 7.15–8.95 ppm. In the ^13^C NMR spectra of the synthesized compound, the resonance at 29.01 and 65.82 ppm were assigned to 3 CH_2_ of propane, while the resonances at m = 109–154 ppm were attributed to C–Ar. Additionally, HRMS confirmed the molecular ion peaks for the compound.

### 3.2 Electrochemical measurement

#### 3.2.1 Potentiodynamic polarization study

**Fig. 5:**
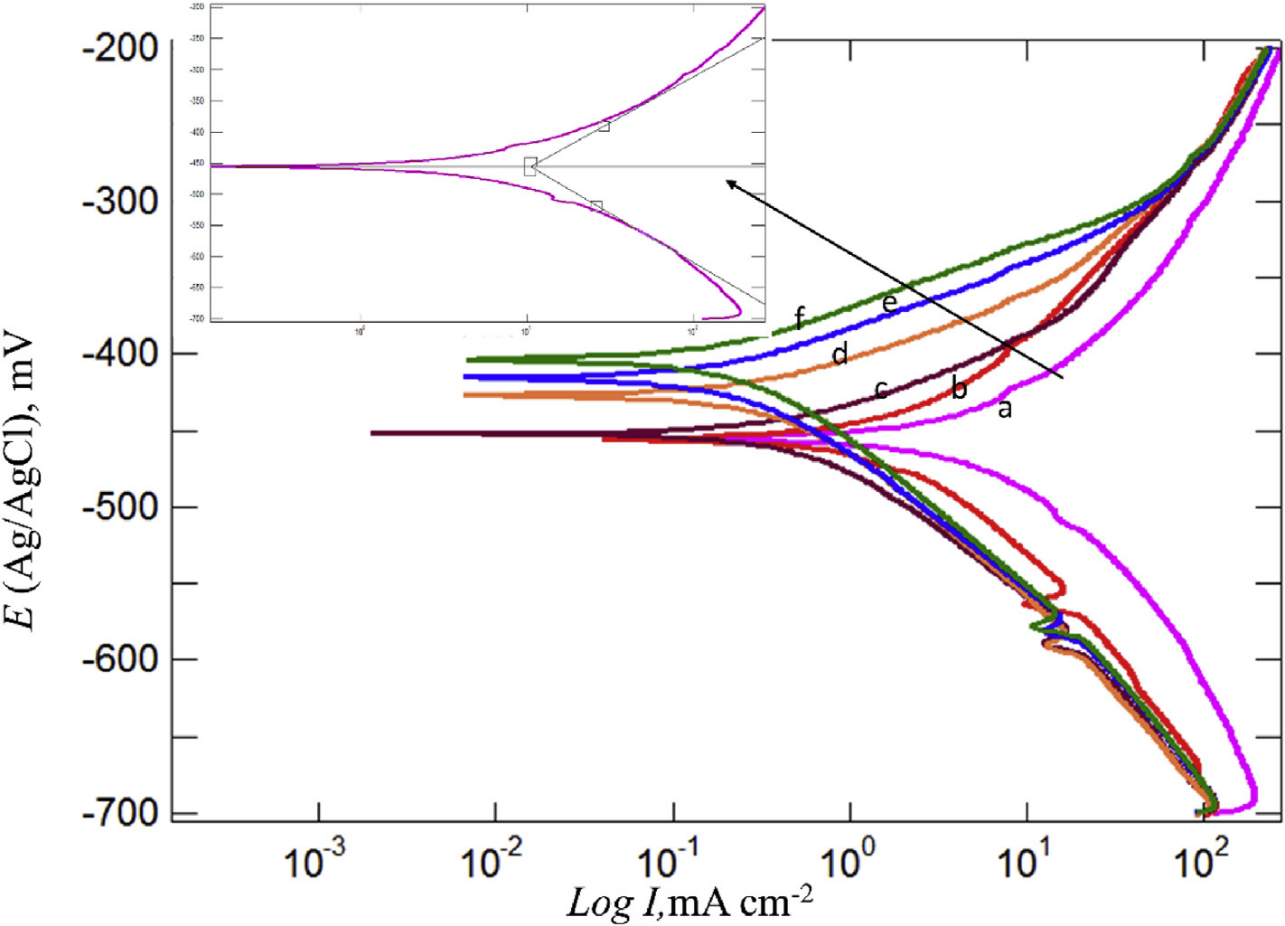
Potentiodynamic polarization curves for a) mild steel in 2 M H_2_SO_4_, and b) 0.05, c) 0.10, d) 0.25, e) 0.50, and f) 1 mM of BQYP molecules at 298 °K.

The polarization behavior of different concentrations of BQYP, which were added to 2 M H_2_SO_4_ solution, is shown in Fig. 5, and the inhibition efficiency (IE%) of each concentration is reported in Table 1. Table 1 indicates that the corrosion current density (*Ι*_corr_) decreased from 6.9531 mA/cm^2^ with blank acid to 0.5731 mA/cm^2^ with the highest concentration of BQYP; *Ι*_corr_ was calculated from extrapolating the linear part of the measured anodic or cathodic curves and intersecting these extrapolated lines with a line corresponding to the corrosion potential *(E*_corr_). Depending on the value of *I*_corr_, the inhibition efficiency increases with increasing BQYP concentration. IE% can be calculated from Eq. (1):

(1) $$IE\%=\left(1-\frac{I_{corr}}{I_{corr}^{o}}\right)\times 100$$

where *Ι*_corr_ and *Ι* ^*o*^_corr_ are the corrosion current with and without BQYP molecules, respectively.

**Table 1:** Electrochemical parameters obtained from Tafel polarization on mild steel in 2 M H_2_SO_4_ in the presence of various concentrations of BQYP solution.

*C*_inh_ (mM)	*E*_corr_ (mV)	*β*_a_ (mV dec^−1^)	*β*_c_ (mV dec^−1^)	*i*_corr_ (mA cm^−2^)	θ	*IE*%
0	–482.29	161.19	175.92	6.95	0.00	0.00
0.05	–457.4	126.47	139.8	3.38	0.51	51.41
0.10	–453.77	116.96	102.38	2.40	0.65	65.42
0.25	–428.58	74.58	114.03	1.09	0.84	84.36
0.50	–416.07	61.17	109.44	0.61	0.91	91.24
1.00	–405.18	55.85	113.6	0.57	0.91	91.76

Based on the different values of *E*_corr_ between the inhibited and uninhibited solution, the BQYP compound is considered a mixed type inhibitor that reduces the anodic metal dissolution as well as the cathodic hydrogen evolution, since the difference did not exceed ±85 mV with increased trends towards the anodic direction. Saranya et al., (2016) explained that the corrosion process might be affected by two mechanisms of adsorption of the inhibitor molecules on the metal surface. The first possible mechanism is the geometrical reduction of the contact reaction area by the blocking effect; the second is the changing of activation energy of the anodic and/or cathodic reaction, which is called the energy effect. It is crucial to predict which mechanism is predominant to control the corrosion process. The theoretical explanation shows that if there is no change of *E*_corr_, the adsorption follows the blocking effect, while if there is a change of *E*_corr_, as a consequence the adsorption follows the energy effect (de Souza and Spinelli, 2009). In this study, the noticeable change of *E*_corr_ demonstrated the adsorption of BQYP molecules on the steel surface, which may have resulted from the energy effect, although the blocking effect could not be completely ignored. The anodic and cathodic Tafel slope is an indication of symmetry of the energy barrier for cathodic and anodic reactions. It was observable that there was no significant change of the shape of the polarization curves with the different ranges of BQYP concentrations, which indicated that the addition of BQYP molecules did not affect the mechanism of steel dissolution or the hydrogen evolution at the cathodic site. Thus, BQYP molecules reduced the *Ι*_corr_ by adsorption on the steel surface. Moreover, each inhibited polarization curve shows a localized minimum *Ι*_corr_ around –600 mV, which may have appeared due to the formation of a passive film of adsorbed inhibitor molecules or an accumulation of corrosion products on the steel surface. The further increase of *Ι*_corr_ was ascribed to passage of the corrosive ion and breakdown of passivation (Sherif et al., 2011).

#### 3.2.2 Electrochemical impedance spectroscopy (EIS)

Another most essential technique to measure inhibition efficiency is EIS, which provides the kinetic information of the system under study. EIS was studied after 30 min to establish the steady-state of the system.

**Fig. 6:**
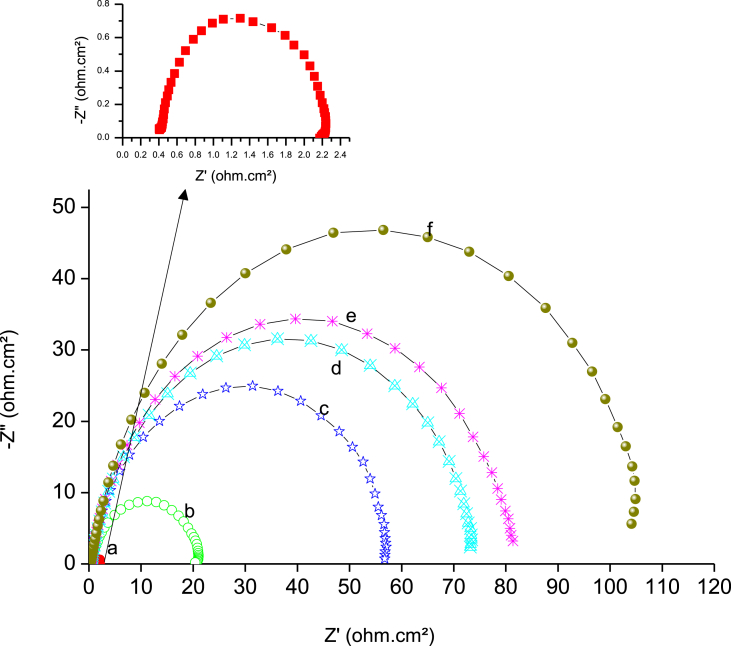
Nyquist plots for a) mild steel in 2 M H_2_SO_4_, and b) 0.05, c) 0.10, d) 0.25, e) 0.50, and f) 1 mM of BQYP molecule at 298 °K.

**Fig. 7:**
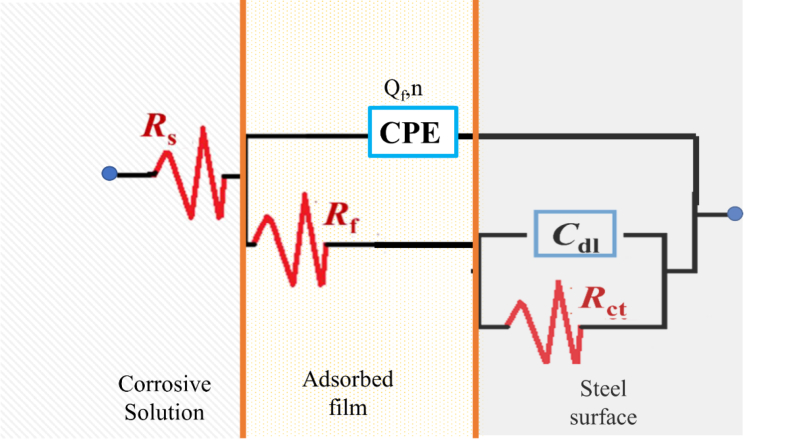
Model of the equivalent circuit.

EIS can represent the Nyquist plot as shown in Fig. 6; it can be observed that the uninhibited system showed one semicircle, indicating that the corrosion of the metal was mainly under activation or charge transfer control. Furthermore, the inhibited system showed a depressing behavior of a semicircle through a capacitive loop at the high range of frequency arising from the charge transfer resistance. This was attributed to the formation of an oxide layer on the steel surface (Wit et al., 1979; Bessone et al., 1983); this protective layer acts as a barrier preventing the dissolution of the metal. The capacitive loop is strong evidence that the corrosion reaction was under activation or charge transfer control. The deviation from the ideal semicircle may arise for many reasons, such as the roughness of the steel surface or the heterogeneity of the dielectrics of the metal (Carranza and Alvarez, 1996). It is also realized that the constant phase element at the low-frequency region of the Nyquist curve originates from the adsorption of the inhibitor molecule on the metal surface (Dagdag et al., 2019). As reported in previous work (Ganash, 2015), the increase of the concentration of inhibitor molecules leads to a more adsorbed molecule on the surface of the metal, which isolates the metal from the corrosive medium and provides more protection. The best equivalent circuit used to estimate the kinetic parameter, depending on the value of Chi-squared (χ^2^ < 1 x 10^−3^), is shown in Fig. 7.

The model (recorded in Table 2) contains solution resistance (*R*s), adsorbed film resistance (*R*_f_), charge transfer resistance (*R*_ct_), the resistance between the steel surface and the outer Helmholtz plane (Saha et al., 2016), the double-layer capacitance (*C*_dl_), which is estimated at maximum frequency of the imaginary axes (*Z*″), and the replacement of the real capacitance constant phase element of the adsorbed film (CPE), which includes the parameter *Q*_f_ and heterogeneity coefficient *n*. Q_f_ represents the magnitude of CPE and n has a value ranging from –1 to 1, where –1 is characteristic of inductive behavior, 0 is characteristic of the resistance, 1 corresponds to the capacitor behavior, and 0.5 is associated with the Warburg impedance (diffusion or mass transfer control).

**Table 2:** Electrochemical parameters obtained from *EIS* on mild steel in 2 M H_2_SO_4_ in the presence of various concentrations of BQYP solution.

*C*_inh_ (mM)	*R*_s_ (Ω cm^2^)	*Q*_f_ (μF cm^−2^)	*R*_f_ (Ω cm^2^)	*C*_dl_ (μF cm^−2^)	*R*_ct_ (Ω cm^2^)	*R*_p_ (Ω cm^2^)	*IE*%
0	0.33	742	1.57	46.5	10.55	12.12	0.000
0.05	0.32	249	1.59	40.6	19.52	21.11	42.58
0.10	0.31	139	19.1	45.9	38.23	57.33	78.86
0.25	0.30	128	6.97	9.90	66.92	73.89	83.59
0.50	0.31	156	0.95	42.9	80.36	81.31	85.09
1.00	0.29	176	3.14	18.8	108.1	111.24	89.11

The diameter of the semicircle represents the charge transfer resistance (*R*_ct_) of blank acid and different concentrations of BQYP. As noted from the figure, *R*_ct_ increases with increasing concentration of BQYP, while the value of Cdl is reduced with increases in concentration of the inhibitor, which arise from two reasons; the first one is the decrease of the free surface area where the solution is existing as a result of the formation of adsorped BQYP film. The second reason is the increased thickness of the adsorbed film due to the replacement of the water molecules by BQYP molecules, which decreases the dielectric constant of the metal and increases the thickness of the double layer (Khaled and Al-Qahtani, 2009). Depending on the Helmholtz equation (Eq. (2)), the double-layer capacitance is inversely proportional to the thickness of the protective layer. Consequently, the increase of the protective layer thickness and/or decrease in the dielectric constant of the adsorbed film leads to reduced *C*_dl_.

(2) $$C_{dl}=\frac{\varepsilon \varepsilon^{o}A}{d}$$

where *ε* is the dielectric constant of the medium, *ε*^o^ is vacuum permittivity, *A* is the area of the electrode, and *d* is the thickness of the adsorbed layer.

For that, the calculated IE% also increases with increasing concentration to reach the maximum value of 89% at 1 mM.

IE% is estimated from Eq. (3).

(3) $$IE\%=\left(1-\frac{R_{p}^{o}}{R_{p}}\right)\times 100$$

where *R*_p_ and *R*^o^_p_ are the polarization resistances with and without BQYP molecules, respectively. However, *R*_p_ is equal to the summation of *R*_f_ and *R*_ct_, (*R*_p_ = *R*_f_ + *R*_ct_).

### 3.3 Adsorption process

The aptitude of inhibitor molecules to adsorb on the surface is considered the main factor to determine IE% of the inhibitor. The degree of coverage mentioned is calculated from Eq. (4):

(4) $$\theta =\left(1-\frac{I_{corr}}{I_{corr}^{o}}\right)$$

**Fig. 8:**
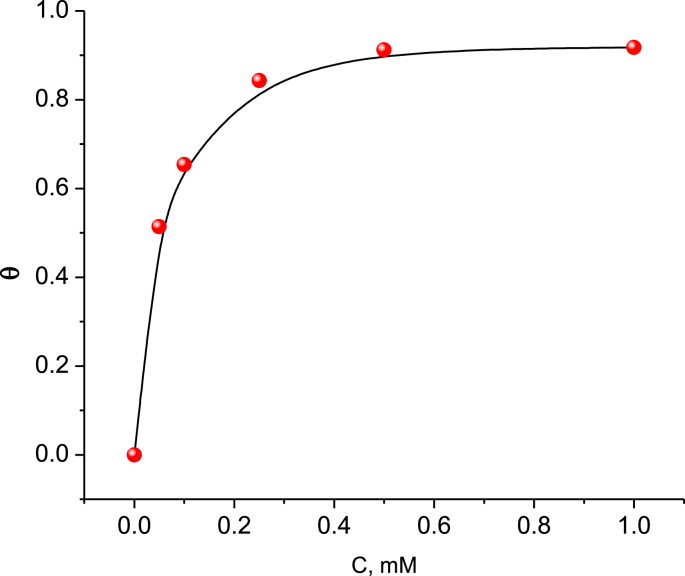
The simple relationship between *θ* and BQYP concentration at 298 °K.

The value of *θ* is reported in Table 1, and the results show that *θ* increased with increasing concentrations of BQYP molecules. The plotting of *θ* against a different concentration of BQYP molecules, as shown in Fig. 8, represented the simple isotherm curve for the adsorption process. The curve appeared as an S-shape, confirming the formation of a complete monolayer of BQYP molecules on the metal surface.

**Fig. 9:**
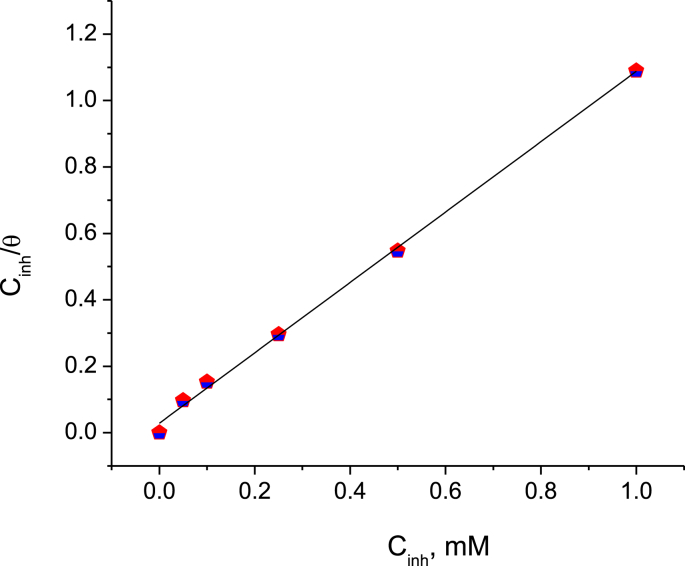
Langmuir Isotherm plot for adsorbed BQYP molecules on mild steel in 2 M H_2_SO_4_ at 298 °K.

The Langmuir isotherm represents the most optimum fitting diagram to experimental data (Langmuir, 1917) with a correlation coefficient R^2^ = 0.998. This model is associated with the adsorption of one layer of the inhibitor molecule on the metal surface and can be represented by Eq. (5):

(5) $$C_{inh}\theta^{-1}=\frac{1}{K_{ads}}+C_{inh}$$

where C_*inh*_ is the concentration of BQYP molecules and *Κ*_ads_ is the adsorption constant, which can be calculated from the straight line in Fig. 9. *K*_ads_ describes the strength of the adsorption process between the metal and the inhibitor molecules. Thus, the large value of *Κ*_ads_ in this study is evidence that BQYP molecules were adsorbed strongly on the steel surface. The other adsorption parameter such as *H*_ads_ and *G*_ads_ can be calculated from Eqs. (6) and (7), respectively:

(6) $$\Delta G_{ads}=-RT\text{ln}\left(55.5K_{ads\;}\right)$$

(7) $$\text{ln}\frac{\theta }{1-\theta }=\text{ln }AC\;\text{inh}-\frac{\Delta H_{ads}}{RT}$$

where *R* is the general gas constant, *Τ* is the absolute temperature, *Α* is an independent constant, and the numerical value 55.5 is the concentration of water in a molar unit at 298 °K at the electrode-electrolyte interface. The adsorption parameter is reported in Table 5. Earlier research described the indication of the sign of *H*_ads_, where a positive value (H_ads_ > 0) was attributed to the chemisorption process (Bentiss et al., 2005), while a negative value (H_ads_ < 0) was attributed to the chemisorption process (Ali et al., 2005), physisorption process (Oguzie et al., 2004), or a mixture of them (Benabdellah et al., 2007). The negative value of Δ*G*_ads_ elucidates the spontaneous adsorption of BQYP molecules on the steel surface. In this study, Δ*G*_ads_ = –18.88 kJ/mol, thus this adsorption was classified as physical adsorption. In 2015, Umoren et al. demonstrated that the value of Δ*G*_ads_ is a useful factor in categorizing the type of adsorption process as chemical _(_Δ*G*_ads_ ≤ –40 kJ/mol)_,_ physical (Δ*G*_ads_ ≥ –20 kJ/mol), or a combination of physical and chemical adsorption (–40 kJ/mol < Δ*G*_ads_ < –20 kJ/mol) (Umoren and Gasem, 2015).

**Fig. 10:**
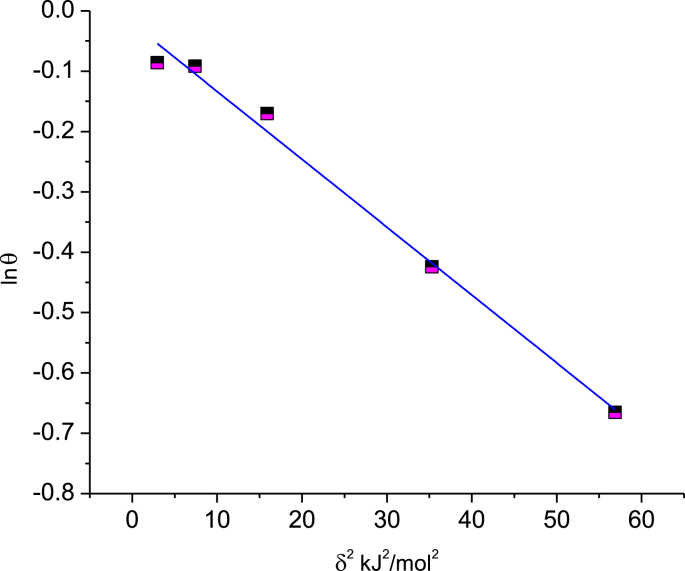
Dubinin–Radushkevich Isotherm plot for adsorption of BQYP molecules on mild steel in 2 M H_2_SO_4_ at 298 °K.

Applying another isotherm called the Dubinin–Radushkevich relationship, which represents the linear relation between ln *θ* and the Polanyi potential *δ* (Eq. (8)), is shown in Fig. 10, since R^2^ = 0.988.

(8) $$\text{ln}\theta =\text{ln}\theta_{max}\;-\beta \delta^{2}$$

where *δ* = *RT*ln(C^−1^_*inh*_ + 1), β is the relation constant, and θmax is the maximum number of inhibitors covering the metal surface. The Dubinin–Radushkevich relationship is considered a well-known model to distinguish the adsorption mechanism as a physical or chemical process depending on the value of the mean energy of adsorption E, which may be calculated from Eq. (9):

(9) $$E=\frac{1}{\sqrt{2\beta }}$$

As reported before (Karahan et al., 2006; Saltalı et al., 2007), when *E* > 8 kJ/mol, the adsorption is classified as chemical adsorption, while when *E* < 8 kJ/mol, physical adsorption is demonstrated. In our case, *E* = 6.7 kJ/mol, which agrees with the BQYP molecule being adsorbed physically on the steel surface.

### 3.4 Effect of temperature

**Fig. 11:**
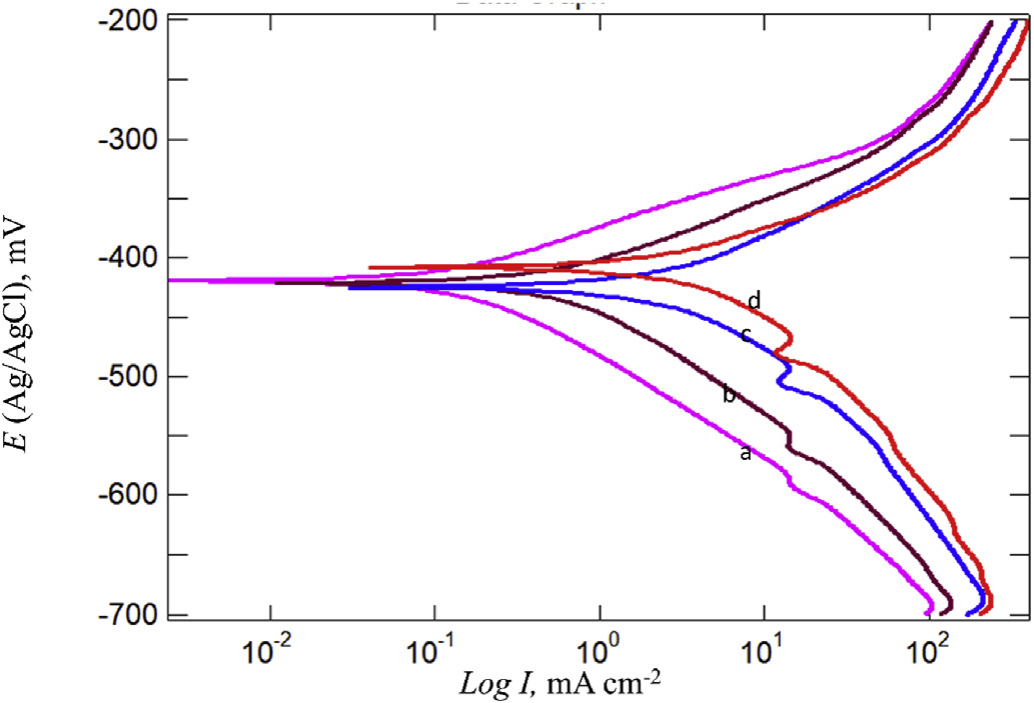
Potentiodynamic polarization curves for mild steel in the presence of 1 mM of BQYP molecules in 2 M H_2_SO_4_ at a) 298, b) 308, c) 318, and d) 328 °K.

**Fig. 12:**
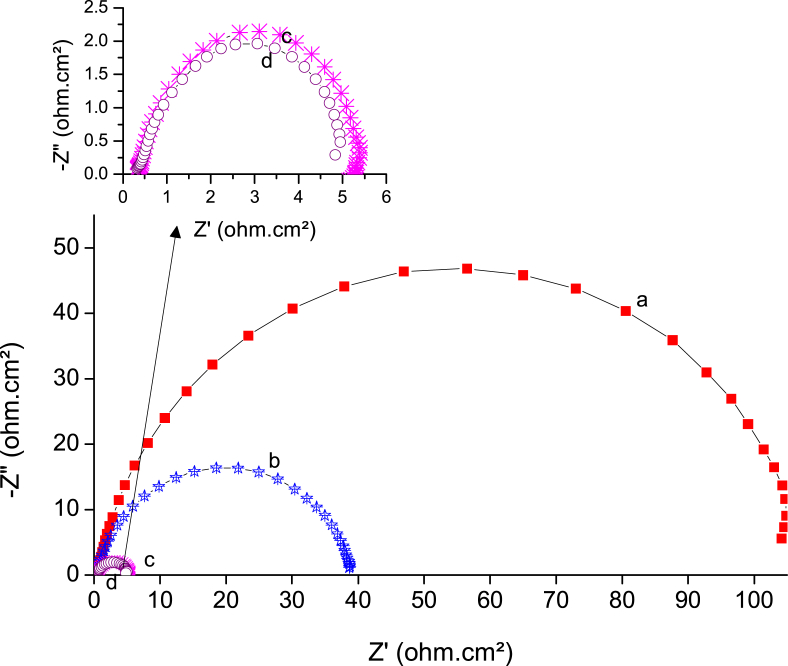
Nyquist plots for mild steel in the presence of 1 mM of BQYP molecules in 2 M H_2_SO_4_ at a) 298, b) 308, c) 318, and d) 328 °K.

To determine the type of adsorption, either chemical or physical, it is essential to change the temperature of the system under study. In this regard, four different temperatures (298, 302, 318, and 328 °K) were used for free acid and inhibited solution using potentiodynamic polarization (Fig. 11) and the EIS technique (Fig. 12). The corrosion parameters for each technique were recorded, as seen in Tables 3 and 4, respectively. Inspection of data in these tables shows that *I*_corr_ increased and *R*_p_ decreased with increasing temperature; this observation is strong evidence of physical adsorption of BQYP molecules. Studying the effects of temperature change is imperative to gaining an understanding of the stability of the adsorbed molecules on the steel surface. Reduced inhibitor performance with increasing temperature may be associated with the short period between the adsorption and desorption process of BQYP molecules, so the steel surface becomes free and exposed to the corrosive solution for a long time.

**Table 3:** The effects of temperature on the polarization parameters in the presence of 1 mM BQYP on mild steel in 2 M H_2_SO_4_.

*T* (°K)	*E*_corr_ (mV)	*β*_a_ (mV dec^−1^)	*β*_c_ (mV dec^−1^)	*i*_corr_ (mA cm^−2^)
298	–405.18	55.85	113.6	0.57
308	–423.14	81.45	139.15	2.04
318	–423.13	104.73	167.27	7.51
328	–466.29	91.79	185.3	10.03

**Table 4:** The effect of temperature on EIS parameters in the presence of 1 mM BQYP on mild steel in 2 M H_2_SO_4_.

*T* (°K)	*R*_s_ (Ω cm^2^)	*Q*_f_ (μF cm^−2^)	*R*_f_ (Ω cm^2^)	*C*_dl_ (μF cm^−2^)	*R*_ct_ (Ω cm^2^)	*R*_p_ (Ω cm^2^)
298	0.29	176	3.14	18.8	108.1	111.24
308	0.30	252	1.29	36.5	37.45	38.74
318	0.32	494	0.62	93.1	4.47	5.09
328	0.36	499	4.61	98.9	4.2	4.20

**Table 5:** Adsorption and Corrosion activation parameters in the presence of 1 mM BQYP on mild steel in 2 M H_2_SO_4_.

Sample	*K*_ads_ (mM)^−1^	Δ*H*_ads_ kJ/mol	Δ*G*_ads_ kJ/mol	*E*_a_ kJ/mol	ΔH* kJ/mol	ΔS* J/K mol	ΔG* kJ/mol			
							298 °K	308 °K	318 °K	328 °K
Free acid	-	-	-	45.36	42.76	–85.42	68.21	69.06	69.94	70.77
inhibitor	35	–54.03	–18.8	80.84	78.25	14.31	73.99	73.85	73.70	73.56

Moreover, the activation energy (E_a_) can be calculated from Arrhenius' equation (Eq. (10))

(10) $$\text{ln}i_{corr}=\text{ln}A-\frac{E_{a}}{RT}$$

where *A* is the Arrhenius constant.

**Fig. 13:**
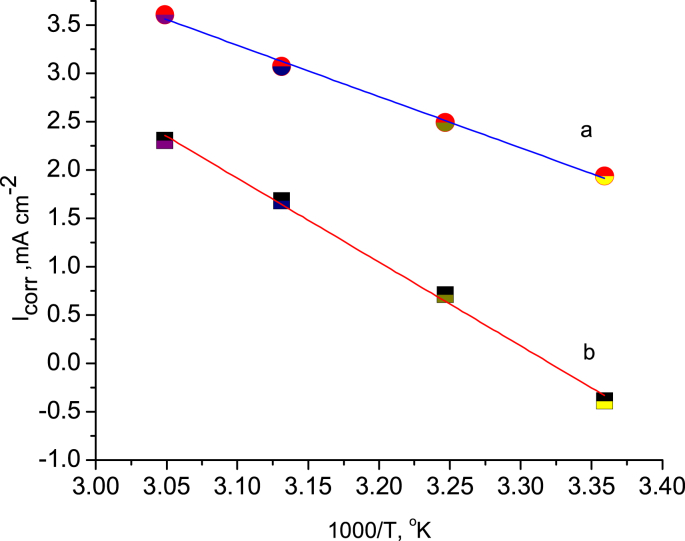
Arrhenius relationship for free H_2_SO_4_ a) without and b) with 1 mM of BQYP molecules.

Plotting ln *I*_corr_ vs 1/*Τ* gives a straight line with a slope equal to –*Ε*_*a*_*/R* as represented in Fig. 13 and listed in Table 5. It is clear from Table 5 that the activation energy *E*a in the presence of BQYP molecules (80.84 kJ/mol) is higher compared to the free acid (45.36 kJ/mol); this confirms that this adsorption is classified as physical adsorption. This may be explained by the increasing formation of the energy barrier due to the adsorbed BQYP molecules at the steel–solution interface, which makes the corrosion reaction challenging to proceed (Noor, 2005).

**Fig. 14:**
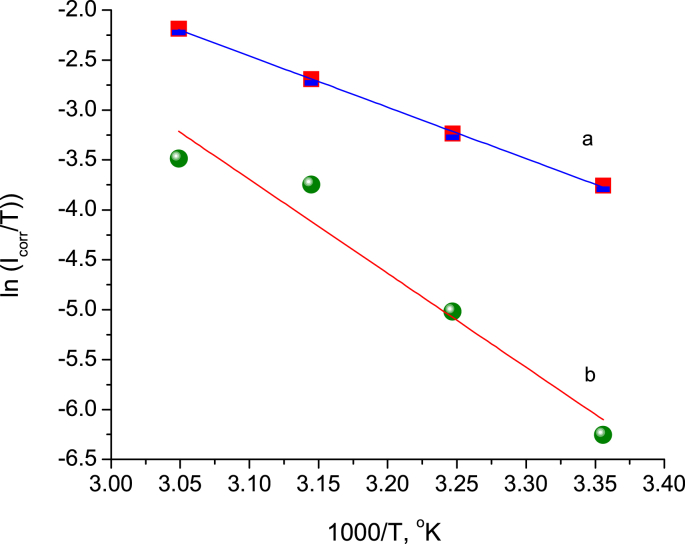
Transition state plots for free H_2_SO_4_ a) without and b) with 1 mM of BQYP molecules.

The activation parameters such as the enthalpy (ΔH*), entropy (ΔS*), and free energy (Δ*G*∗) of activation can be calculated from Eqs. (11) and (12), as shown in Fig. 14, and the results of all activation parameters are given in Table 5. The positive sign of ΔH* implies an endothermic reaction. As observed in Table 5, ΔS* increased for the inhibited system compared to the uninhibited system. The result shows that the corrosion process proceeded from more orderly for an uninhibited solution (ΔS* = –85.42 J/K mol) to random in the presence of an inhibitor (ΔS* = 14.31 J/K mol). A similar finding was previously reported for a study of the corrosion behavior in acidic media such as some N-heterocyclic compounds (Noor, 2005), thiol compounds (Makhlouf and Wahdan, 1995), and some cyclic amine compounds (El-Awady, Abd-El-Nabey, and Aziz 1992). However, the negative value of ΔS* for the free acid explained that the formation of activated complexes in the rate-determining step required the association process rather than the dissociation process. The high positive value of ΔG* of the inhibited system compared to the uninhibited blank solution emphasized that the stability of the activated compound decreased in the presence of BQYP molecules.

(11) $$\text{ln}\left(\frac{i_{corr}}{T}\right)=\left[(\text{ln}\left(\frac{R}{Nh}\right)+\left(\frac{\Delta S^{*}}{R}\right)\right]-\frac{\Delta H^{*}}{RT})$$

(12) $$\Delta G^{*}=\Delta H^{*}-T\Delta S^{*}$$

where *N* is Avogadro's number, and *h* is Planck's constant.

## 4 The theoretical calculation

The quantum study using the DFT method at B3LYP/6 + 311G (d,p) was used to calculate the quantum parameters that are related to inhibition protection of BQYP molecules, which are listed in Table 6.

**Table 6:** Quantum chemical parameters in eV for BQYP molecules.

Molecule	*E*_HOMO_	*E*_LUMO_	Δ*E*	*E*_I_	*E*_A_	χ	*ξ*	*ϕ*	Δ*N*	Δ*E*_T_
BQYP	–5.919	–1.439	4.481	5.919	1.439	3.679	2.240	0.446	0.7413	0.560

**Fig. 15:**
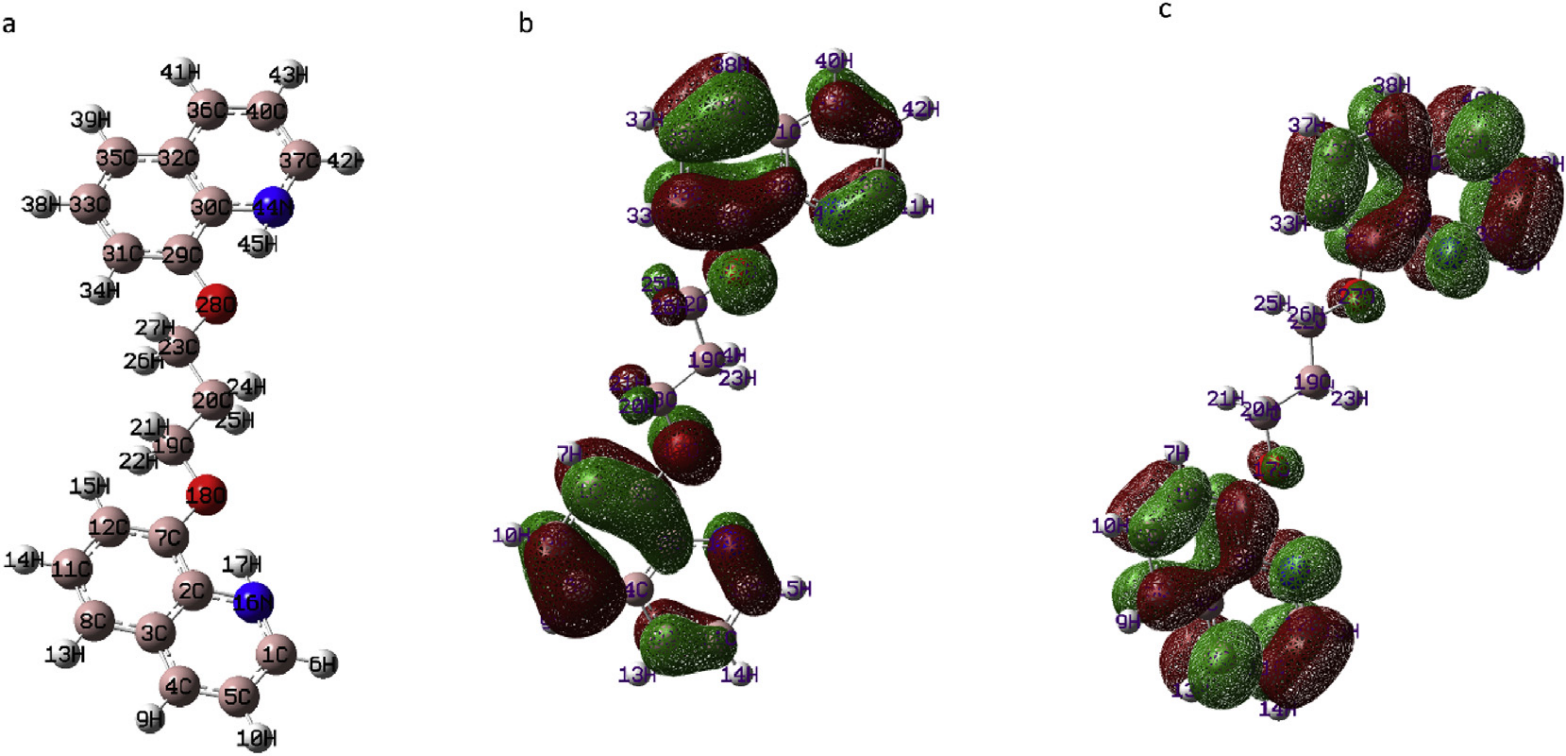
a) The optimized geometric structures of BQYP molecules. (b) The geometric structure of the HOMO orbital and c) LUMO orbital.

As described previously (Gao and Liang, 2007), the interaction between the metal surface and the inhibitor molecule was mainly ascribed to the donation electron from the high occupied orbital (HOMO orbital) of the inhibitor molecule to the vacant d-orbital of the metal. Furthermore, the interaction between the metal and the inhibitor molecule can be due to acceptance of an electron from the d-orbital of the metal to the lower unoccupied orbital (LUMO orbital), depending on the high energy value of the HOMO orbital (*E*_HOMO_ = –5.919 eV), which indicates the good tendency to donate an electron. In other words excellent inhibition efficiency is provided due to the high ability of adsorption of the inhibitor molecule on the metal surface (Robert G. Parr, László v. Szentpály, and Shubin Liu 1999). Conversely, the lower energy value of the LUMO orbital (*E*_LUMO_ = –1.439 eV) specifies the excellent ability to accept electrons and improve the adsorption process of BQYP molecules on the steel surface. In general, the adsorption process of the inhibitor increases with increasing *E*_HOMO_ and decreasing *E*_LUMO._ The HOMO and LUMO orbitals, as well as the optimized structural geometry are depicted in Fig. 15.

The energy difference (Δ*E*) between *E*_LUMO_ and *E*_HOMO_ is considered a significant factor in describing the adsorption of the inhibitor on the metal surface. Δ*E* can be calculated from Eq. (13)

(13) $$\Delta E=E_{LUMO}-E_{HOMO}$$

It was reported that the small value of Δ*E* is strong evidence of inhibition ability (Tang et al., 2009). In this study (Δ*E* = 4.481 eV), the energy value, was very low, which indicated that BQYP afforded proper protection.

Calculated quantum parameters such as electron affinity (*E*_A_), ionization energy (*E*_I_), electronegativity (χ), and absolute hardness and softness (*ξ* and *ϕ*j, respectively) can be calculated from Eq. (14) (Pearson, 1988), (15) (Parr and Pearson, 1983), and (16) (Pearson, 1988), respectively.

(14) $$x=\frac{E_{I}+E_{A}}{2}$$

(15) $$\xi =\frac{E_{I}-E_{A}}{2}$$

(16) $$\phi =\frac{1}{\xi }$$

where *E*_A_ = –*E*_LUMO_ and *E*_I_ = –*E*_HOMO_. From the theory of hard and soft acid–base, the metal will be softer in acidic solutions, and the inhibitor molecule will have more softness and lower hardness than the inhibitor in the neutral solution. Thus, the softer molecule will be adsorbed easily on the metal surface.

The low value of *ξ* and the high value of ϕ are measurements of the chemical stability of the inhibitor molecule, which enhances the adsorption process (Ebenso et al., 2010a, Ebenso et al., 2010b). The computed fraction of electron transfer from BQYP molecules to the steel surface (Δ*N*) was estimated from Eq. (17).

(17) $$\Delta N=\frac{x_{Fe}-x_{inh}}{2\left(\xi_{Fe}+\xi_{inh}\right)}$$

where $x_{Fe}$(7 eV) and $\xi_{Fe}$ (0 eV).

Ju et al. suggested that the value of Δ*N* > 0 indicates the transfer of an electron from a molecule to the metal, while Δ*N* < 0 indicates the transfer of an electron from metal to the molecule. It has also been reported that the inhibitor protection or the electron-donating ability increases when Δ*N* < 3.6 (Zucchi, 2001). Thus, the positive value of Δ*N* is strong evidence of electron sharing between BQYP molecules and the steel surface. However, the high value of Δ*N* corresponded with increases in the inhibitor ability. It is worth noting that Δ*N* refers to the ability to donate electrons rather than the exact number of transfer electrons (Obot et al., 2015).

The total energy (Δ*E*_T =_ –ξ/4) is associated with the acceptance of an electron on the active center and release back of the electron from the same center (Gómez et al., 2006). It was reported (Ebenso et al., 2010a, Ebenso et al., 2010b) that when Δ*E*_T_ < 0 and ξ > 0, the transfer of the charge from or to the inhibitor molecule is enabled.

**Fig. 16:**
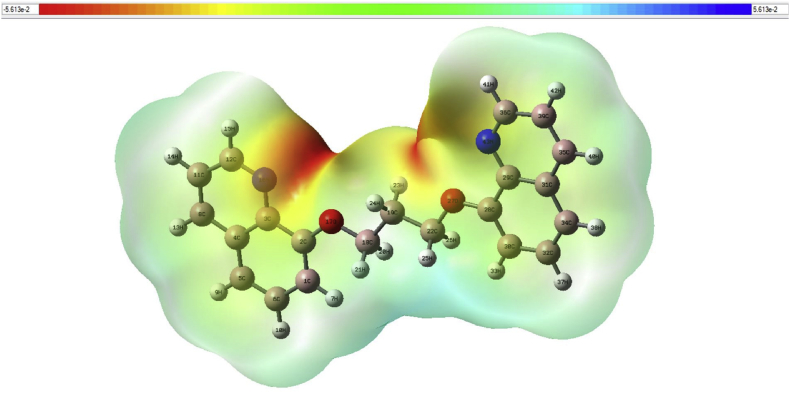
The molecular electrostatic map of BQYP molecules.

From the molecular electrostatic map, as shown in Fig. 16, the negative zone delocalized on the heteroatoms O and N, while the positive region delocalized on C and H atoms. This map confirms that the heteroatoms, i.e., the electronegative atoms, represent the active center of the BQYP molecule. In this regard, the steel surface represents the electroactive center.

**Fig. 17:**
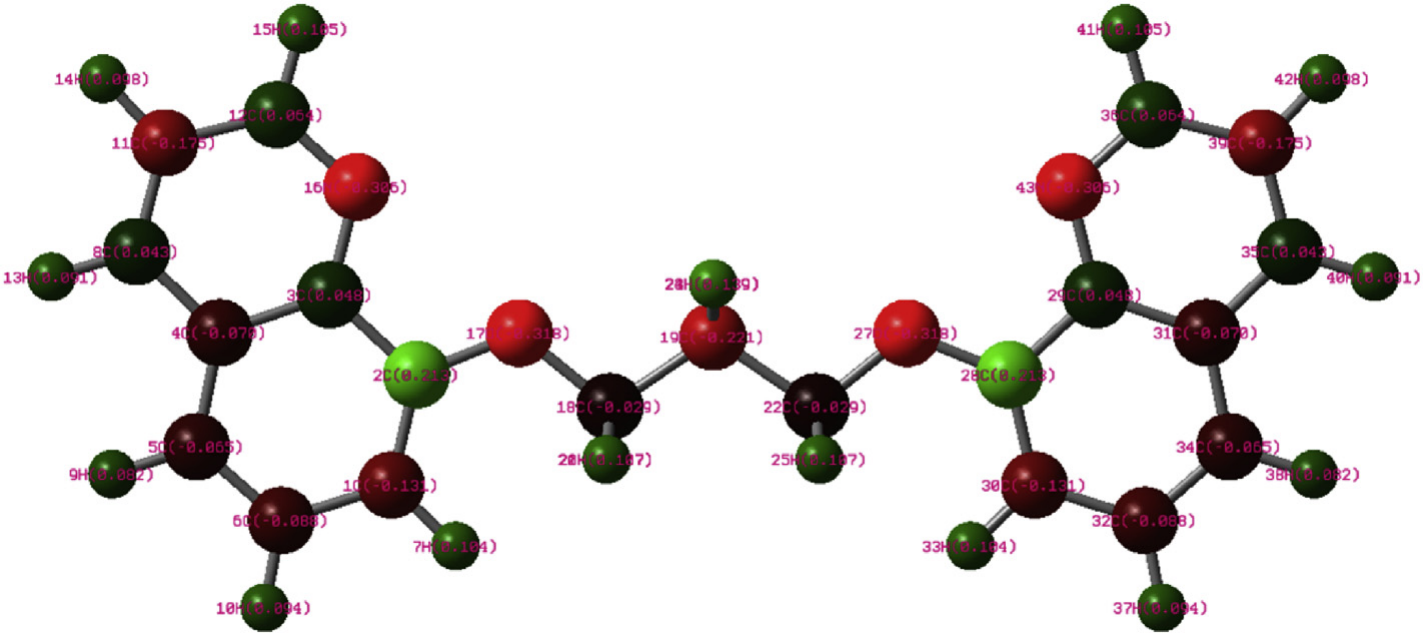
The Mulliken population charge analysis of BQYP molecules.

Mulliken population charge analysis (Fig. 17) was utilized to predict the most favorable atom to interact with a steel surface. The hetero atoms with the highest negatively charge preferably adsorb onto the steel surface through the donor-acceptor process. It was clear that atoms N16 (–0.305949), O17 (–0.318217), O27 (–0.318219), and N43 (–0.305949) represent the active nucleophilic center, indicating that the adsorption process of BQYP molecules on the steel surface was carried out among these atoms.

## 5 Explanation of inhibitors' work

The structure of the inhibitor is considered the main factor affecting the adsorption process; BQYP molecules contain atoms with lone pair electrons (O and N) as well as π-electrons in the phenyl ring. BQYP presents as a protonated molecule in acidic media through oxygen and nitrogen atoms.

BQYP + xH ↔ [BQYP-H]^x+^

As is well-known, the steel metal gains a positive charge in H_2_SO_4_ acid due to the dissolution of the metal. Based on this fact, Khamis et al., (2013) suggested the anion of electrolyte adsorbed firstly on the positively charged metal, and at the same time, it attracts the protonated inhibitor molecule. In other words, the anion works as a bridge connecting both positive sides and able to form a thin layer of BQYP molecules on the steel surface (physical adsorption), which prevents the direct contact of steel with the corrosive acid and proved the required protection. Doner et al. (Döner and Kardaş, 2011) suggested that the protonated inhibitor molecules are adsorbed on the cathodic site on the metal surface, which suppresses the hydrogen evolution as the cathodic reaction, consequently, reduces the corrosion reaction.

## 6 Conclusions

The most notable finding to appear from this report is that BQYP molecules reduce the corrosion rate of mild steel to a lower value and provide excellent inhibition efficiency in highly corrosive 2 M H_2_SO_4_ acid reaching 91.7%. The BQYP molecules were physically adsorbed on the steel surface. Regarding the increase in the value of the activation parameter of the inhibited solution compared to the uninhibited solution, the polarization measurement emphasized that the BQYP acts as a mixed type inhibitor, which can hinder both anodic and cathodic reactions but trends more toward the anodic direction. Finally, the inhibitory effect of BQYP molecules on the steel surface in highly concentrated acidic medium was successfully provided by applying the DFT approach for the theoretical quantum calculation, and the theoretical calculation indicates that BQYP molecules interact with the steel surface via donor-acceptor interactions.

## Declarations

### Author contribution statement

Aisha Ganash, Zahra M. Alamshany: Conceived and designed the experiments; Performed the experiments; Analyzed and interpreted the data; Contributed reagents, materials, analysis tools or data; Wrote the paper.

### Funding statement

This work was supported by the Deanship of Scientific Research (DSR), King Abdulaziz University, Jeddah, under grant No (D-1441-175-247).

### Competing interest statement

The authors declare no conflict of interest.

### Additional information

No additional information is available for this paper.
